# Altered Cerebral Blood Flow One Month after Systemic Chemotherapy for Breast Cancer: A Prospective Study Using Pulsed Arterial Spin Labeling MRI Perfusion

**DOI:** 10.1371/journal.pone.0096713

**Published:** 2014-05-09

**Authors:** Kelly N. H. Nudelman, Yang Wang, Brenna C. McDonald, Susan K. Conroy, Dori J. Smith, John D. West, Darren P. O'Neill, Bryan P. Schneider, Andrew J. Saykin

**Affiliations:** 1 Department of Medical and Molecular Genetics, Indiana University School of Medicine, Indianapolis, Indiana, United States of America; 2 Training in Research for Behavioral Oncology and Cancer Control Program, Indiana University School of Nursing, Indianapolis, Indiana, United States of America; 3 Center for Neuroimaging, Department of Radiology and Imaging Sciences, Indiana University School of Medicine, Indianapolis, Indiana, United States of America; 4 Indiana Alzheimer Disease Center, Indiana University School of Medicine, Indianapolis, Indiana, United States of America; 5 Indiana University Melvin and Bren Simon Cancer Center, Indiana University School of Medicine, Indianapolis, Indiana, United States of America; 6 Department of Medicine, Indiana University School of Medicine, Indianapolis, Indiana, United States of America; Hangzhou Normal University, China

## Abstract

Cerebral structural and functional alterations have been reported after chemotherapy for non-CNS cancers, yet the causative mechanism behind these changes remains unclear. This study employed a novel, non-invasive, MRI-based neuroimaging measure to provide the first direct longitudinal measurement of resting cerebral perfusion in breast cancer patients, which was tested for association with changes in cognitive function and gray matter density. Perfusion was measured using pulsed arterial spin labeling MRI in women with breast cancer treated with (N = 27) or without (N = 26) chemotherapy and matched healthy controls (N = 26) after surgery before other treatments (baseline), and one month after chemotherapy completion or yoked intervals. Voxel-based analysis was employed to assess perfusion in gray matter; changes were examined in relation to overall neuropsychological test performance and frontal gray matter density changes measured by structural MRI. Baseline perfusion was not significantly different across groups. Unlike control groups, chemotherapy-treated patients demonstrated significantly increased perfusion post-treatment relative to baseline, which was statistically significant relative to controls in the right precentral gyrus. This perfusion increase was negatively correlated with baseline overall neuropsychological performance, but was not associated with frontal gray matter density reduction. However, decreased frontal gray matter density was associated with decreased perfusion in bilateral frontal and parietal lobes in the chemotherapy-treated group. These findings indicate that chemotherapy is associated with alterations in cerebral perfusion which are both related to and independent of gray matter changes. This pattern of results suggests the involvement of multiple mechanisms of chemotherapy-induced cognitive dysfunction. Additionally, lower baseline cognitive function may be a risk factor for treatment-associated perfusion dysregulation. Future research is needed to clarify these mechanisms, identify individual differences in susceptibility to treatment-associated changes, and further examine perfusion change over time in survivors.

## Introduction

Breast cancer treatment has made great strides, leading to an overall five-year survival rate of 89% according to SEER [1]. Given this high survival rate, resulting side effects are an area of increasing concern. Cognitive dysfunction in particular has been associated with breast cancer, chemotherapy, radiation, and anti-estrogen treatments, underscoring the need to clarify the mechanisms of disease and treatment effects on cognition [2]–[15].

Neuroimaging has been used to measure structural and functional effects of cancer and chemotherapy treatment, including treatment-associated frontal gray matter density (GMD) decrease in breast cancer patients [16]–[18]. However, characterization of the full spectrum of cerebral alterations associated with chemotherapy is far from complete; one novel area of investigation is the effect of chemotherapy on resting cerebral perfusion using pulsed arterial spin labeling (PASL) magnetic resonance imaging (MRI). PASL is a noninvasive advanced technique capable of measuring blood flow by using magnetically labeled arterial blood water as an endogenous contrast tracer. This technique can provide quantitative, stable, and physiologically meaningful images [19]. It has been shown to be well-suited to longitudinal studies of cerebral perfusion in healthy and diseased individuals, or as a surrogate marker of metabolism [20]. Based on previous prospective breast cancer studies, observed GMD decrease post-treatment suggests the likelihood of accompanying hypoperfusion; however, patterns of increased and decreased activation observed during cognitive task performance post-treatment might indicate the presence of hyperperfusion as well [16]–[18], [21]–[22]. To test these competing hypotheses, we conducted the first controlled prospective study of cerebral perfusion in breast cancer patients treated with (Ctx+) and without (Ctx−) standard-dose chemotherapy and healthy controls (HC) using PASL MRI. We predicted that the Ctx+ group would evidence statistically significant changes in brain perfusion compared to the HC and Ctx− control groups post-treatment, in response to or in order to compensate for chemotherapy-induced cellular, vascular, or other tissue damage, and that this change would correlate with cognitive performance. Additionally, we analyzed perfusion changes for associations with previously reported frontal GMD change to investigate the functional independence of these measures and underlying mechanisms [18].

## Methods

### Ethics Statement

All participants gave written informed consent according to the Declaration of Helsinki under a protocol approved by the Indiana University Institutional Review Board.

### Overall Study Design

This manuscript focuses on a subset of data from a prospective, longitudinal investigation of cancer and treatment-related cognitive effects which includes a comprehensive study protocol of neuroimaging, neurocognitive, and behavioral assessments as well as biomarker investigation. Other aspects of this and a related cohort have previously been reported [17], [18], [21], [23]. In brief, study participants include Ctx+ and Ctx− female breast cancer patients and demographically matched HC. For Ctx+ patients, all study measures were acquired at baseline (after surgery but before radiation, chemotherapy, and/or anti-estrogen treatment), approximately one month after chemotherapy treatment completion, and approximately one year later. These time points were chosen to allow examination of both sub-acute and longer-term effects of treatment, with a particular focus on the effects of chemotherapy. Ctx− patients and HC were studied at yoked intervals (see Table 1). In addition to the PASL and T1 sequences examined for this report, the detailed MRI examination included PD/T2 and FLAIR structural sequences, working and episodic memory functional MRI tasks, magnetic resonance spectroscopy, and diffusion tensor imaging. Neurocognitive assessment included a targeted research battery with emphasis on episodic memory and executive functioning, as well as measures briefly assessing other neuropsychological domains (e.g., general intellectual ability, language, attention, processing speed, visuospatial and motor skills, etc.). Self-report measures included demographic and medical information, treatment sequelae, self-perceived cognition, mood and anxiety, and diet and lifestyle factors. Finally, blood samples were acquired for routine labs, genetic analyses, and banked for future investigation of potential biomarkers. Blood work was typically acquired in the early morning, while other study measures were acquired over the course of one or two study visits depending on participant preference. MRI scanning was conducted prior to cognitive assessment whenever possible, though timing of study measures varied depending on scheduling availability.

**Table 1:** Sample Demographics.

	Ctx+	Ctx−	HC	*P* a
	(N = 27)	(N = 26)	(N = 26)	
Age at Baseline (yrs.)	49.9 (7.6), (36–69)	52.0 (8.9), (31–68)	48.4 (10.1), (32–69)	0.350
Education (yrs.)	15.4 (2.8), (10–20)	15.4 (2.5), (11–20)	15.6 (2.1), (12–20)	0.954
Estimated Full Scale IQ (Barona Index) [49]	110.1 (6.7), (89–117)	111.1 (6.2), (98–117)	111.2 (5.2), (99–116)	0.760
Handedness (R,L/Amb)	26,1	24,2	24,2	0.662
White, Non-Hispanic	77.8%	88.5%	80.8%	0.588
CES-D raw score: Baseline	10.9 (9.7), (0–38)	9.0 (8.8), (0–34)	7.2 (7.6), (0–28)	
CES-D raw score: 1 M	14.6 (9.3), (2–33)	9.8 (9.7),(0–40)	6.9 (7.0),(0–25)	0.216
STAI-S raw score: Baseline	35.1 (15.0), (20–78)	28.9 (8.0), (20–47)	30.9 (10.2), (20–55)	
STAI-S raw score: 1 M	35.4 (12.4), (20–62)	32.2 (13.0), (20–64)	32.4 (12.4), (20–62)	0.601
Inter-scan interval (days)	158.7 (68.9), (73–387)	177.2 (72.2), (106–480)	159.6 (28.0), (116–253)	0.465
Cancer stage: 0 (DCIS)	0	6		
Cancer stage: I	11	18		0.000
Cancer stage: II	12	2		
Cancer stage: III	4	0		
Received radiotherapy	2	16		0.000
On anti-estrogen therapy^b^: Baseline	0	1 TAM		
On anti-estrogen therapy: 1 M	1 ANA, 1 TAM, 1 LET	11 TAM, 1 TAM/LEU, LET, 2 ANA, 1 EXE, 1 RAL		
Chemotherapy Regimen c ^,^ d				
DOX/CYC/paclitaxel	8			
DOC/CYC	8			
DOC/carboplatin	6			
DOC/DOX/CYC	2			
DOC/cisplatin	1			
DOX/CYC	1			
paclitaxel	1			

Values are Mean (Standard Deviation), (Range).

1 M =  one month post chemotherapy completion (or yoked intervals).

a P value for one-way ANOVA with treatment groups for age, education, IQ, handedness, ethnicity, interscan interval, and radiotherapy; group-by-time analysis with treatment group for CESD and STAI, chi-square with treatment group for cancer stage.

b ANA =  anastrozole; TAM =  tamoxifen, LET =  letrozole; EXE =  exemestane; RAL =  raloxifene; LEU =  lueprolide acetate.

c Nine Ctx+ patients were also treated with trastuzumab, one was also treated with sunitinib, and one was also treated with bevacizumab.

d DOX =  doxorubicin, CYC =  cyclophosphamide, DOC =  docetaxel.

### Participants

The study cohort consisted of 27 Ctx+ patients, 26 Ctx− patients, and 26 HC. Only patients with non-invasive (stage 0) or non-metastatic invasive (stages I, II, or III) disease were included; additional exclusion criteria for all participants included prior cancer, substance abuse, and other medical, neurological, and psychiatric risk factors which might affect cerebral structure or function, as described in McDonald et al., 2013 [18]. Based on these criteria, 213 individuals were determined to be ineligible during phone screening. Another 15 individuals were excluded after enrolling in the study due to subsequent determination of ineligibility (e.g., tissue expanders, claustrophobia, etc.), withdrawal, or loss to follow-up, and 18 more individuals were excluded from this analysis due to lack of relevant data or scan quality, yielding 79 eligible participants with required data.

All Ctx+ patients were treated with common standard-dose chemotherapy regimens. Eight neoadjuvant chemotherapy patients' baseline measures were completed before surgery as well as chemotherapy; these individuals did not differ significantly from the other Ctx+ patients for any demographic factors, depression, or anxiety. Patients were recruited via the Indiana University Melvin and Bren Simon Cancer Center recruitment core and affiliated clinical practices. Demographically matched healthy controls were recruited via community advertisements.

Demographic and treatment characteristics are summarized in Table 1. The Center for Epidemiologic Studies-Depression Scale (CES-D) and the State Trait Anxiety Inventory-State subscale (STAI-S) were used to measure depressive symptoms and anxiety at each visit [24], [25]. Self-reported caffeine consumption on scan days was also obtained. Demographic and treatment variables were assessed for statistical significance using SPSS 19 (SPSS Statistics 19, IBM Corporation, Somers, NY), using ANOVA, general linear models, and chi-square analyses as appropriate.

### MRI Acquisition

Scans were acquired on a Siemens Tim Trio 3T whole-body MRI scanner using a 12-channel receiver-only head coil. Subjects were scanned in a conscious resting state with closed eyes. Cerebral perfusion measurements were obtained using a previously published scan protocol [19]. Briefly, a Q2TIPS PASL sequence using the PICORE labeling scheme was applied. Utilizing a 10 cm labeling region with 25 mm spacing from the distal edge of the labeled region to the image section, an adiabatic inversion pulse for labeling was followed by optimized inversion time delays TI_1_ = 700 ms and TI_2_ = 1800 ms, chosen so as to minimize intravascular signal intensity at 3T. Images were acquired using a gradient-echo single shot EPI readout, with acquisition parameters: TR/TE = 3000/13 ms, FOV = 224 mm, and matrix  = 64×64. The imaging region consists of 16 contiguous ascending axial slices of 7 mm thickness. Each perfusion measurement consists of 100 dynamic (50 control and label image pairs) plus one M_0_ image (the equilibrium brain tissue magnetization used to normalize the difference perfusion map) with a scan time of approximately 5 minutes. The scanner's built-in 3D online prospective acquisition correction (PACE) was used to minimize head motion artifact during acquisition. A high resolution T1-weighted magnetization prepared rapid gradient echo (MPRAGE) image and a high resolution EPI whole brain scan were acquired for subsequent reference and normalization; T2-weighted and fluid attenuated inversion recovery (FLAIR) sequences were also acquired to examine for incidental pathology.

### Image Analysis

PASL scan processing was performed using previously published methods [14]. In brief, labeled images were subtracted from matched control images to create a perfusion-weighted time series; these were used to create quantitative regional perfusion maps for each scan, which were normalized to Montréal Neurological Institute (MNI) space in SPM8 (Wellcome Department of Cognitive Neuroscience, London, UK), resampled to 2 mm^3^ voxels, and smoothed with a FWHM kernel of 6×6×8 mm. Image analyses in SPM8 were run with and without age at baseline and scan-day caffeine consumption as covariates given previous findings of perfusion variance [26], [27]. However, inclusion of these variables is not shown as groups were balanced and these variables did not significantly alter any results.

A general linear model approach was utilized to conduct statistical parametric mapping on a voxel-by-voxel basis in SPM8. To specifically examine perfusion in gray matter, the Pick Atlas gray matter mask was used as an explicit mask in all analyses [28]. MRI image acquisition and analysis of GMD was performed as described in McDonald et al., 2013 [18].

Baseline analyses using a random effects model were performed to identify voxels with initial perfusion differences between groups. Weighted contrast vectors were entered for each group in the design matrix as described in McDonald et al., 2013 [18]. To test for areas in which cancer patients showed hypoperfusion relative to controls at baseline, −1 was entered in the Ctx+ and Ctx− baseline columns, while 2 was entered in the control baseline column. Alternate directionality and other interactions were similarly tested. The voxel-wise critical significance threshold (*P*_crit_) was set to 0.001 uncorrected, with a minimum cluster extent (k) of 100 voxels for this and subsequent tests to reduce noise.

An unbiased F test of overall effects was performed to identify any statistically significant perfusion differences between groups and times. The cluster of voxels with the most significant perfusion difference was used to graph group-by-time contrast estimates and 90% confidence intervals. All groups were tested alone for perfusion change over time using weighted contrast vectors. Based on these results, the Ctx+ group was analyzed for perfusion increase over time relative to HC in an interaction model, also using weighted contrast vectors.

### Perfusion and Cognition Analysis

Due to the small cohort size, we chose to investigate cognition utilizing an overall neuropsychological performance test score (global score), generated by averaging scores for eight neuropsychological domains in a similar fashion as in Conroy et al., 2013 [29] and Ahles et al., 2008 and 2010 [3], [4]. To discern whether chemotherapy was associated with a change in cognition, global score change was assessed for association with treatment group in SPSS 19 using a one-way ANOVA. Based on the results, global baseline score was chosen for testing with Ctx+ perfusion change. We extracted baseline and post-treatment cluster scores for the right precentral gyrus (area of significant increase in Ctx+ compared to HC) using MarsBar in SPM8, and subtracted baseline from post-treatment scores to obtain a change score for each Ctx+ individual [30]. Ctx+ perfusion change scores were tested for Pearson correlation with global baseline scores in SPSS.

### Perfusion and GMD Change Association Analysis

To test for association of GMD and perfusion, we compared the previously identified frontal GMD change clusters with the more regionally diffuse pattern of change observed for perfusion. Association was only tested in the Ctx+ group, since only this group had statistically significant changes in both measures. To analyze this association, we extracted the two frontal clusters where significant GMD decreases occurred after chemotherapy using MarsBar in SPM8 and averaged them to obtain one GMD measure for each subject at each time point [30]. The measures at baseline were subtracted from the post-treatment measures, yielding a change score for each individual. These GMD change scores were used as the covariate of interest in a multiple regression testing for association with whole brain perfusion change. Perfusion change was measured by subtracting PASL baseline scans from post-treatment scans using the ImCalc utility in SPM8 to create new scans only including regions of change. After multiple regression association testing of frontal GMD change with whole brain perfusion change in SPM8, again using the Pick Atlas gray matter mask, positive and negative associations were analyzed. Covarying for age did not significantly alter the results and is not reported. To graph GMD and perfusion, statistically significant perfusion change clusters from the positive association were extracted using MarsBar and averaged to one change score per individual; these were plotted against the GMD change measure. One individual's perfusion and GMD scores were more than three standard deviations below the mean. Inclusion/exclusion of this individual's scores did not alter the statistical significance of the findings, so these data points are excluded from the graphical presentation of the results.

## Results

### Demographic and Baseline Comparisons

Ctx+ patients had significantly higher stage disease than Ctx− patients (X^2^ = 18.82, df = 3, *P*<0.001), as expected given current treatment protocols, and most had not received either radiotherapy or hormone therapy by one month post-chemotherapy treatment. There were no other statistically significant demographic differences or group-by-time interactions with depression or anxiety symptoms (CES-D, STAI-S, Table 1). The second scan occurred on average six months after the baseline visit; interscan intervals did not differ significantly between groups (Table 1). Eight patients received neoadjuvant treatment; these individuals did not demonstrate statistically significant demographic differences or group-by-time interactions with depression or anxiety symptoms compared to adjuvant Ctx+, Ctx−, or HC groups.

Baseline comparisons indicated no significant between-group differences in perfusion.

### Perfusion Change: Chemotherapy Group Hyperperfusion

**Figure 1:**
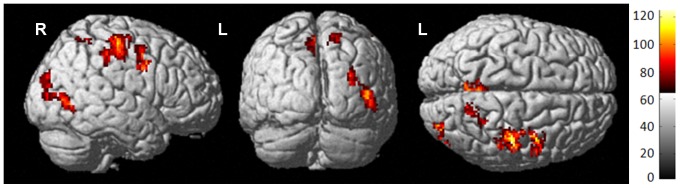
Group-by-time analysis (F test). Surface rendering of perfusion differences from baseline to one month post-treatment for all groups (*P*_crit_<0.001 uncorrected, k = 100). Colored areas indicate statistically significant changes between groups and/or times; red to yellow color scale indicates increasing statistical significance, with yellow areas indicating the most significant regions.

**Figure 2:**
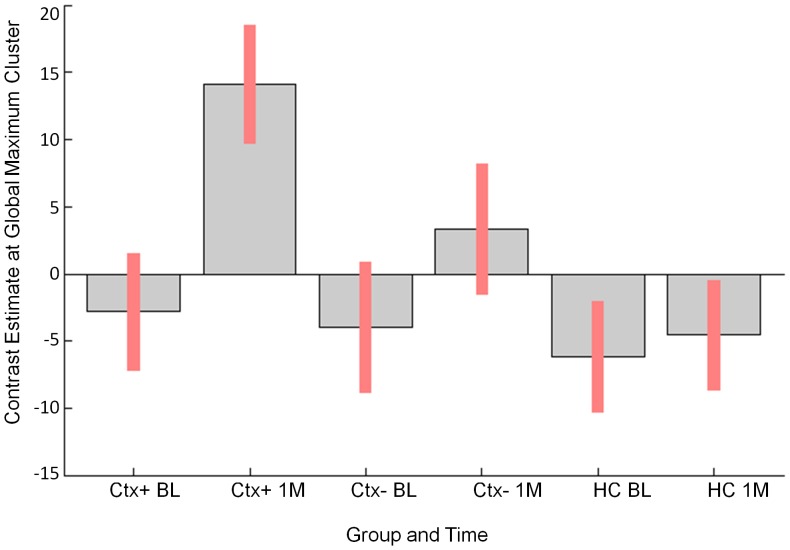
Maximum perfusion change. Right postcentral gyrus perfusion signal from overall F test graphed by group and time (Red bars indicate 90% confidence intervals (C.I.)). Key: BL =  baseline, 1 M =  one month post-treatment.

An F test across all groups and both time points indicated statistically significant perfusion change over time, primarily in the right hemisphere (Figure 1, Table 2). The cluster of maximal change, located in the right postcentral gyrus, is presented for all groups and both time points (Figure 2). In this region, the Ctx+ group showed hyperperfusion at one month post-treatment relative to both comparison groups. For this and subsequent image analyses, results including MNI coordinates, cluster extents, multiple comparison-corrected *P* values, T and Z scores, and regions are presented in Tables 2–5.

**Table 2:** Regional Perfusion Changes for the Overall F Test, All Groups by Times.

x^a^	y^a^	z^a^	k^b^	T	Z	P_FWE−corr_ c	Region description (for cluster peak)
44	−24	56	437	8.32	4.87	0.009	R Postcentral G (BA3)
16	−58	54	112	7.91	4.72	0.018	R Precuneus (BA7)
48	−70	2	268	7.78	4.67	0.022	R M Temporal G (BA37)
42	0	48	183	7.74	4.65	0.024	R Precentral G (BA6)
−10	−46	50	264	6.39	4.10	0.217	L Precuneus (BA7)

a MNI coordinates.

b Cluster extent.

c Peak-level p value.

Key: R =  Right, L =  Left, G =  Gyrus, BA =  Brodmann Area, I =  Inferior, M =  Middle, S =  Superior.

**Table 3:** Regional Perfusion Changes for Ctx+ Post-Treatment Increase.

x^a^	y^a^	z^a^	k^b^	T	Z	P_FWE−corr_ c	Region description (for cluster peak)
46	−22	56	673	5.32	5.09	0.012	R Postcentral G (BA3)
32	−84	24	1464	5.20	4.98	0.000	R S Occipital G (BA19)
66	−24	34	116	5.03	4.83	0.552	R Postcentral G (BA2)
−14	12	−10	119	4.27	4.14	0.540	L Lentiform Nucleus
−6	−86	26	425	4.26	4.14	0.059	L Cuneus (BA19)
−6	−64	56	207	4.22	4.10	0.282	L Precuneus (BA7)
−16	−94	−16	101	4.20	4.08	0.612	L I Occipital G (BA17)
−54	−18	54	128	4.07	3.96	0.507	L Postcentral G (BA3)

a MNI coordinates.

b Cluster extent.

c Cluster-level p value.

Key: R =  Right, L =  Left, G =  Gyrus, BA =  Brodmann Area, I =  Inferior, M =  Middle, S =  Superior.

**Table 4:** Regional Perfusion Changes for Ctx+ Increase Relative to Controls.

x^a^	y^a^	z^a^	k^b^	T	Z	P_FWE−corr_ c	Region description (for cluster peak)
40	−24	60	151	4.22	4.10	0.429	R Precentral G (BA4)

a MNI coordinates.

b Cluster extent.

c Cluster-level p value.

Key: R =  Right, L =  Left, G =  Gyrus, BA =  Brodmann Area, I =  Inferior, M =  Middle, S =  Superior.

**Table 5:** Regional Perfusion Changes for Ctx+ Perfusion and GMD Positive Association.

x^a^	y^a^	z^a^	k^b^	T	Z	P_FWE−corr_ c	Region description (for cluster peak)
12	18	−24	578	10.13	6.33	0.006	R Rectal G (BA11)
−50	−60	−22	153	9.71	6.20	0.317	L Fusiform G (BA37)
44	48	−10	136	8.26	5.68	0.377	R M Frontal G (BA11)
−4	50	−24	285	7.61	5.43	0.084	L Rectal G (BA11)
22	44	50	513	7.34	5.31	0.011	R S Frontal G (BA8)
−42	−58	44	248	7.20	5.25	0.120	L I Parietal Lobule (BA40)
34	−40	56	264	6.85	5.09	0.103	R Postcentral G (BA40)
−8	58	40	255	6.71	5.03	0.112	L S Frontal G (BA9)
−38	42	38	189	6.52	4.49	0.219	L S Frontal G (BA9)
−48	−58	44	114	6.10	4.73	0.470	L I Parietal Lobule (BA40)
38	64	12	608	5.92	4.64	0.005	R M Frontal G (BA10)
24	−10	−12	121	5.74	4.54	0.439	R Amygdala
−22	−8	0	159	5.07	4.17	0.298	L Lentiform Nucleus

a MNI coordinates.

b Cluster extent.

c Cluster-level p value.

Key: R =  Right, L =  Left, G =  Gyrus, BA =  Brodmann Area, I =  Inferior, M =  Middle, S =  Superior.

**Figure 3:**
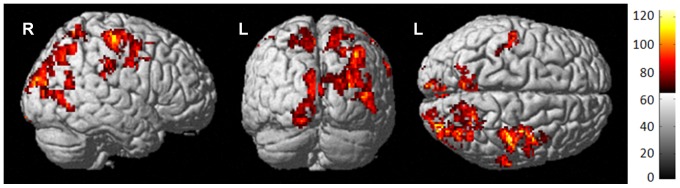
Ctx+ group post-treatment hyperperfusion. Surface rendering of Ctx+ increase from baseline to one month post-treatment indicates that bilateral superior and posterior brain regions demonstrate hyperperfusion post-treatment.

**Figure 4:**
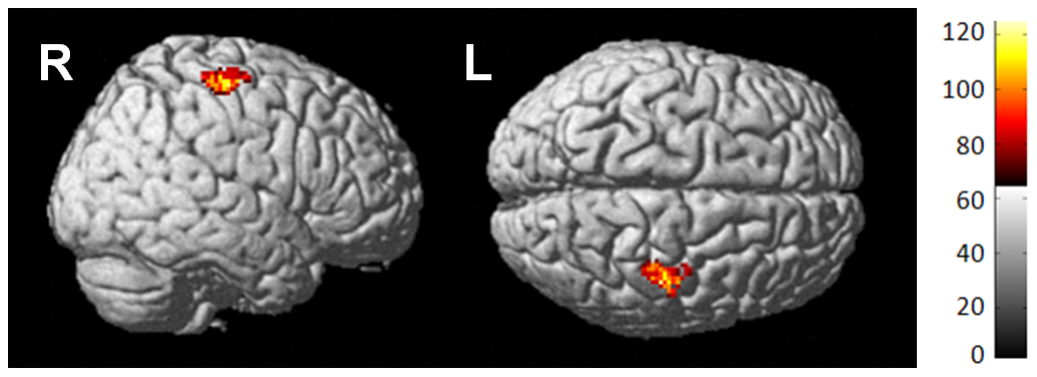
Ctx+ perfusion increase compared to HC. Surface rendering of Ctx+ increase compared to HC over time indicates statistically significant perfusion increase in Ctx+ in the right precentral gyrus post-treatment.

To further elucidate group differences in change over time, each group was tested individually. Only the Ctx+ group had statistically significant perfusion change, demonstrating an increase in perfusion from baseline to one month post-treatment, primarily in superior and posterior brain regions (Figure 3, Table 3). To determine how much of this increase was statistically significantly different than controls, we analyzed group-by-time interactions; results indicated that the Ctx+ group perfusion increased relative to HC specifically in the right precentral gyrus after treatment, indicating that these patients had resting state hyperperfusion discernible by MRI compared to controls (Figure 4, Table 4). Table 6 lists mean perfusion for both time points as well as perfusion change for the right precentral gyrus cluster.

**Table 6:** Right Precentral Gyrus Perfusion Group Means.

	Ctx+ (N = 27)	Ctx− (N = 26)	HC (N = 26)	*P* a
Cluster Perfusion: Baseline	35.0 (10.7), (18.1–53.7)	33.9 (13.3), (2.5–57.5)	35.9 (10.4), (18.1–57.7)	0.821
Cluster Perfusion: 1 M	49.7 (18.9), (21.5–96.6)	38.8 (16.5), (−2.6–81.7)	33.9 (10.6), (12.8–56.8)	0.002
Cluster Perfusion: Change^b^	15.0 (16.7), (−9.6–57.2)	4.9 (14.7), (−22.1–33.9)	−2.0 (9.0), (−20.9–11.5)	0.000

Values are Mean (Standard Deviation), (Range). 1 M =  one month post-treatment.

a Significance, one way ANOVA with treatment group.

b Change  = 1 M – Baseline.

The eight neoadjuvant patients were not analyzed separately, as the study design was not powered to detect subgroup effects. However, in the neoadjuvant-treated participants the mean perfusion increase in the right postcentral gyrus was almost identical to that observed for the adjuvant Ctx+ participants and Ctx+ group as a whole. The three Ctx+ patients who received anti-estrogen treatment in this interval also displayed a post-chemotherapy perfusion increase, though there was insufficient power for a formal assessment of hormonal effects or interactions. Given that only two Ctx+ patients received radiotherapy treatment during this interval, this factor is also unlikely to contribute significantly to the observed variation.

### Chemotherapy-Associated Hyperperfusion and Cognition

**Figure 5:**
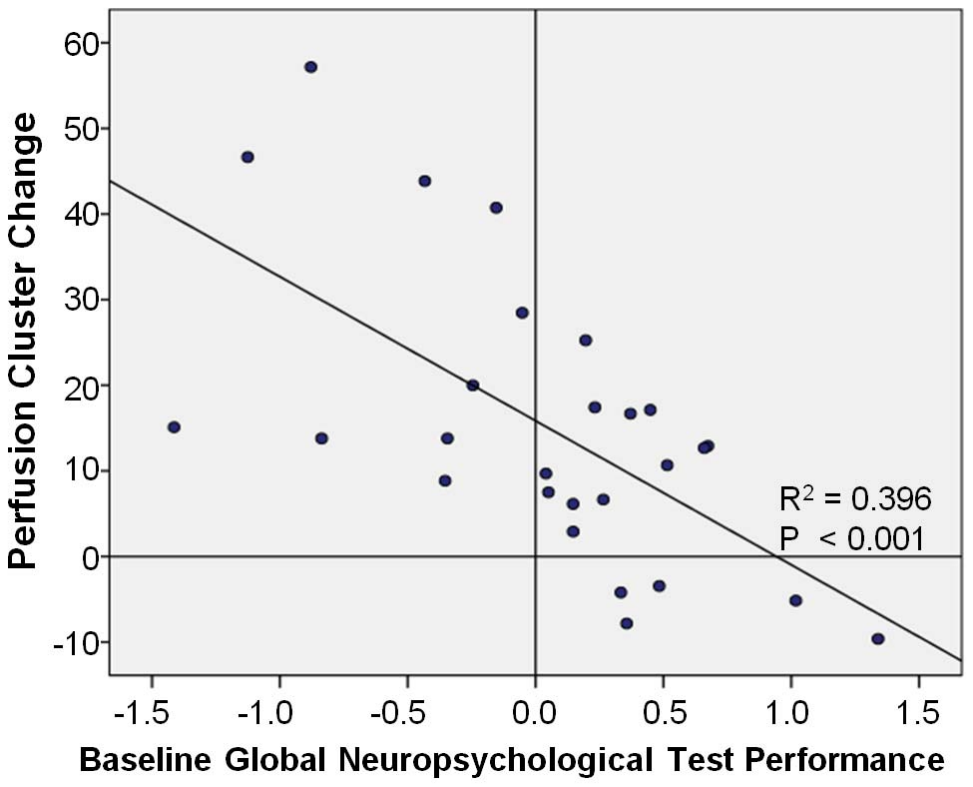
Ctx+ baseline cognitive performance and perfusion change correlation. Individual baseline global neuropsychological test performance (x-axis) graphed with right precentral gyrus perfusion cluster (statistically significant increase in Ctx+ vs. HC from baseline to post-treatment) change from baseline to one month post-treatment, indicating a significant negative correlation of baseline cognition with perfusion change.

To clarify the clinical significance of this perfusion increase, we assessed perfusion change for correlation with cognitive performance. Change in the global neuropsychological test score from baseline to post-treatment was not statistically significantly different between groups, although all groups showed an increase over time likely attributable to practice effects (Table 7). In the Ctx+ group, baseline global score was negatively correlated (r = −0.629, *P*<0.001) with right precentral gyrus perfusion change from baseline to one month (Figure 5), indicating that individuals with lower baseline cognitive performance showed greater perfusion increase over time.

**Table 7:** Global Cognitive Performance.

	Ctx+ (N = 27)^a^	Ctx− (N = 25)^a^	HC (N = 26)	*P* b
Global score: Baseline	0.1 (0.6), (−1.–1.3)	0.2 (0.6), (−1.6–1.0)	0.0 (0.7), (−1.7–1.0)	0.8
Global score: 1 M	0.2 (0.6), (−1.0–1.4)	0.3 (0.6), (−1.3–1.3)	0.2 (0.7), (−1.8–1.3)	0.8
Global score change^c^	0.1 (0.2), (−0.2–0.9)	0.2 (0.2), (−0.2–0.5)	0.2 (0.2), (−0.1–0.8)	0.6

Values are Mean (Standard Deviation), (Range). 1 M =  one month post-treatment.

a Due to missing neuropsychological data, a global score could not be calculated for one Ctx+ individual at 1 M (1 M Ctx+ N = 26), as well as one Ctx− individual at either time point.

b Significance, one way ANOVA with treatment group.

c Change  = 1 M – Baseline.

### Perfusion and GMD Association

**Figure 6:**
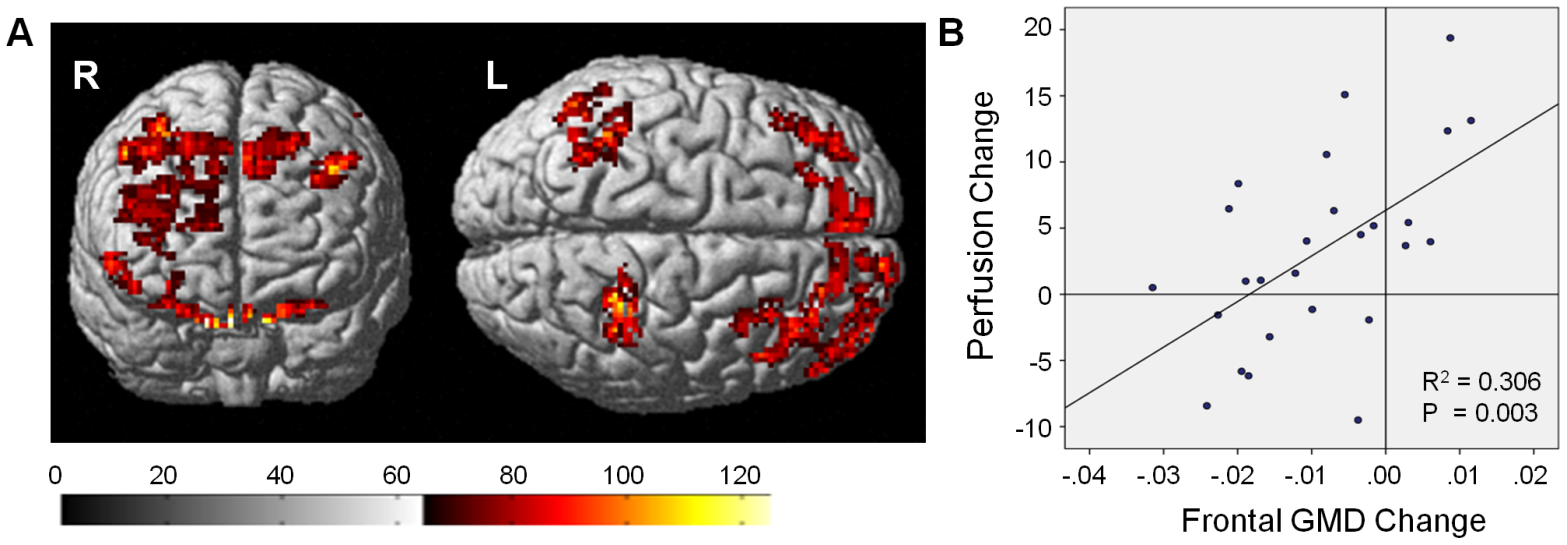
Perfusion and GMD positive association. A) Surface rendering of positive association between frontal GMD decrease and bilateral frontal and parietal perfusion change; association does not overlap with regions of Ctx+ hyperperfusion. B) Frontal GMD decrease (x-axis) graphed with average of all positively associated clusters of perfusion change (y-axis); decreased GMD is associated with decreased primarily frontal perfusion.

Because the chemotherapy-associated perfusion increase was not located in the frontal regions where GMD decrease was observed by McDonald et al., 2013 [18], we hypothesized that these effects might be independent. When the frontal GMD decrease was analyzed for association with whole brain perfusion change, we observed that there were statistically significant regions of bilateral frontal and parietal perfusion change positively correlated with GMD change (Figure 6, Table 5), indicating that decreased frontal GMD was associated with lower perfusion in these regions (r = 0.553, *P* = 0.003). However, if the frontal GMD decrease was associated with the hyperperfusion observed in Ctx+ patients, we would expect to see significant negative association in the superior and posterior regions. In this analysis there were no statistically significant regions of negative association, supporting our hypothesis that these are independent effects.

## Discussion

This is the first prospective, longitudinal study documenting the effects of chemotherapy on resting cerebral perfusion in breast cancer patients. Given conflicting evidence from other measures of cerebral effects of chemotherapy, we investigated two competing hypotheses, chemotherapy-induced hypo- or hyper-perfusion. Although previous evidence suggests that aspects of the cancer disease process and/or host factors in cancer patient may induce cerebral structural and functional alterations, in the present study there were no statistically significant between-group perfusion differences at baseline [18], [21], [29]–[33]. This initial observation suggests that breast cancer itself is not associated with changes in cerebral perfusion. Although requiring replication and possibly specific to the present cohort, the lack of baseline differences suggests that treatment-associated findings are unlikely to be confounded by cancer-induced changes. This is important to note, as it may suggest that perfusion is a more specific indicator of cerebral alterations after chemotherapy, as other functional neuroimaging measures have shown differential alterations both prior to and after chemotherapy.

Although perfusion and metabolism are normally coupled, a previous cerebral resting state metabolic study using fluorodeoxyglucose [^18^F] positron emission tomography (PET) by Silverman et al., 2007 did not find significant differences between control and adjuvant chemotherapy-treated groups, though they did identify a significant correlation between metabolism and the Rey-Osterrieth Complex Figure delayed recall performance in chemotherapy-treated subjects [22]. They also found differential perfusion in Ctx+ group patients compared to controls during a functional [^15^O] PET study. Using functional MRI, McDonald and colleagues found differential activation in Ctx+ group patients, further supporting the idea that chemotherapy may alter perfusion and activation [21]. Thus it appears that PET and MRI may be complementary detection methods for perfusion and metabolic changes in response to chemotherapy. This new PASL data adds another perspective to prior findings, indicating that perfusion is altered in the resting state as well as during tasks, and may be related to previously identified metabolic, cognitive, and structural changes in breast cancer patients.

To date, the precise mechanisms underlying chemotherapy-induced cognitive alterations are unknown. In addition to possible direct neurotoxic injury, several pathways that could influence cerebral structure and function have been posited as potentially affected by chemotherapy, including immune response, DNA repair, oxidative stress, and altered hormone levels or signaling, all of which may be genetically modulated [34]–[36]. Although only small amounts of chemotherapeutic agents have been measured in the brain, this may still be enough to provoke various cellular responses including inflammation and oxidative stress, less effective DNA repair, and possibly cell death, leading to structural and functional alterations and cognitive dysfunction [37]–[41]. All chemotherapy drugs utilized in this cohort are alkylating agents, anthracyclines, platinating agents, or taxanes; these antineoplastic agents target various aspects of DNA repair and cell cycle division, and cause apoptosis. A systemic response to cell death could include several of the mechanisms listed above, and have widespread indirect cerebral effects, such as hyperperfusion. Interestingly, paclitaxel and docetaxel have been shown to have peripheral anti-angiogenic effects, and doxorubicin and paclitaxel have been associated with vascular toxicity [42]–[44]. Since all but one patient received paclitaxel or docetaxel, and only one patient in the Ctx+ group was on bevacizumab during the study, we could not draw any conclusions on the effects of anti-angiogenesis on perfusion; however, the individual receiving bevacizumab did experience increased perfusion post-treatment, consistent with other Ctx+ patients. The study was also not powered to examine any specific vascular toxicity related to doxorubicin or paclitaxel; this will be an important factor to consider in future studies. Although chemotherapy-altered estrogen signaling is still a possible causal mechanism, changes were likely not related to anti-estrogen treatments or aromatase inhibitors, since only three individuals out of 27 in the Ctx+ group were taking these types of medications at one month post-treatment [45]. Although this study was not powered to assess these individuals as a separate group, they did display hyperperfusion that was greater than that observed for the Ctx− or HC groups, though not as large as the Ctx+ group increase as a whole. Based on this evidence, we conclude that anti-estrogen or aromatase inhibitors are unlikely to contribute significantly to the observed hyperperfusion. Radiotherapy was similarly different between groups, with nearly all Ctx+ patients receiving this treatment following the one month post-chemotherapy time point, whereas the majority of Ctx− received radiotherapy before this time point. This emphasizes the specificity of the chemotherapy effect on cerebral perfusion, as radiotherapy is unlikely to confound these results. Finally, caffeine consumption is known to influence cerebral blood flow. We only obtained general self-reported caffeine consumption on scan days, and so were not powered to do a detailed analysis of the variance accounted for by this measure; however, basic self-reported caffeine consumption was not significantly different between groups, suggesting that this was not a confounding factor for this analysis.

Our observation that the statistically significant Ctx+ increase in cerebral perfusion was negatively correlated with baseline neuropsychological test performance suggests that baseline cognitive reserve may indicate an indirect protective mechanism; perhaps the Ctx+ post-treatment perfusion increase is an unsuccessful compensatory mechanism that is most pronounced in individuals with lower cognitive ability [4]. Future research should focus on identifying the biological mechanism driving the potential protective effect of baseline cognitive performance/reserve.

The statistically significant pre- and postcentral gyri regions of chemotherapy-associated cerebral perfusion increase reported here were independent of frontal GMD decreases noted in this cohort. However, bilateral frontal and parietal decreases in perfusion were observed that correlated with GMD decreases. These regional perfusion alterations may be influenced by different mechanisms. This is supported by previous investigations in Alzheimer's disease suggesting that PASL and MRI GMD measures are complementary and have similar sensitivity; consequently if these measures were associated we would likely have detected this in our study [46]. Assuming different mechanisms are involved, one reason for the lack of correlation could be the influence of individual demographic and risk factors on susceptibility; for instance, it may be that some individuals may have genetic polymorphisms impairing drug clearance, leading to toxicity, cell death, and hypoperfusion, while others are physiologically predisposed to inflammation, which might lead to hyperperfusion. The observed lack of association indicates that there are some individuals who experience both hyperperfusion and decreased GMD post-treatment; future studies should examine whether these two measures have additive relationships with cognitive performance or complaints, which may perhaps explain why only a subgroup of breast cancer survivors appear to experience long-term cognitive dysfunction.

Further studies should also examine possible correlations of perfusion with other imaging methods, in order to further clarify the mechanistic basis of chemotherapy-induced cognitive alterations, with the long-term goal of developing preventative measures or treatments targeting these effects. While some cognitive alterations have been observed in survivors of breast cancer years after treatment, other studies show patient improvement by one year post-treatment [4], [17], [21], [47]. If brain changes such as those observed in this study persist, they may be contributing to long-term cognitive sequelae, prioritizing perfusion as a target for therapeutic measures [48]. Future studies should utilize multimodal imaging and long-term prospective designs to clarify the mechanistic basis and persistence of this adverse effect, to help determine the focus of therapeutic efforts and pharmaceutical intervention in this patient population. Additional research could also use animal models to investigate the association of mechanisms such as direct neurotoxicity and immune response with neuroimaging measures; findings from such studies could provide important cues for therapeutic efforts.

This first study of cerebral perfusion in a prospective cohort of breast cancer patients and healthy controls provides evidence that chemotherapy is associated with alterations in cerebral perfusion, independent of cancer effects. We found statistically significant hyperperfusion in superior and posterior regions after chemotherapy, which was not seen in patients who did not receive chemotherapy or controls. While this hyperperfusion was independent of frontal GMD decrease after chemotherapy, regional frontal and parietal hypoperfusion post-treatment did correlate with GMD decreases in these patients. The regional dissociation between hyperperfusion and GMD reduction suggests the involvement of independent functional mechanisms, as well as potential influence of individual risk factors, providing important information to guide future investigation towards therapeutic and preventative strategies.
